# Removal of an Ovarian Tumor

**Published:** 1853-07

**Authors:** G. W. Bayless

**Affiliations:** Hazlewood, Mo.


*Removal of an Ovarian Tumor.* By G. W. Bayless, M. D. of Hazlewood, Mo.—In a letter, addressed to Prof. Pope, the writer says: "When I last saw you, I told you that I had another ovarian case, and expected to operate on my return home. I have recently, (the 15th of January,) operated and lost my patient: and as there are some points of interest in the case, I doubt not a brief account of it would be acceptable to you. Except a few of the leading facts in the general history of the case, I will give only its surgical history.

The patient was of small stature, of very delicate form, fair complexion and light hair; she was twenty years of age, married, and the mother of two children, the younger of which is five and the older seven. About four years ago, she was observed by her friends to be enlarged, and was regarded by them as pregnant. So that the disease may be said to have been of between four and five years standing.

I first saw her in August last, on account of a supposed uterine polypus. She had none. Her abdomen was very much enlarged, and from the history of the case I thought it ovarian disease. I tapped her as a *means of diagnosis.* [I will remark here, that, from my observation in four cases of multilocular ovarian disease, *tapping* is an important *means* of diagnosis. Generally one cyst outgrows the rest, and its accumulated fluid so distends the abdomen as that the tumor or tumors cannot be felt. When the fluid is drawn off, the character of the disease is easily recognised through the laxed abdominal walls.] Such was the result of tapping in this case, as well as the others referred to. Some four or five gallons of fluid were drawn off.

The general health of the patient at this time was tolerably good, and the tapping relieved the only difficulty —slight embarrassment of respiration; no inflammatory symptoms followed the tapping; but in six or seven weeks the fluid had re-accumulated. This was the only time she was tapped.*

Early in November, she took a severe cold, which prevented my operating at that time. By the middle of January, she had recovered from the cold, and her general health was pretty good. I considered the case a very favorable one for the operation; 1st. Because of the *spare, delicate* person, as less liable to have inflammation. 2ndly. The patient had been tapped but once, and the operator would probably not have extensive adhesions to encounter. The result proved the correctness of this latter expectation, and probably also of the former.

The *operation* was commenced by tapping, for the purpose of reducing the size of the tumor. *Three gallons* of straw colored serum were drawn off. The patient suffered no exhaustation from this, and was at once placed on the table. She was put under the influence of chloroform, which acted well. The incision was carried from just below the umbilicus to within two inches of the pubis, and was six inches long. The tumor, upon which the abdominal walls retracted immediately after the tapping, presented itself at the opening, but so large as to require further reduction in size before it could be brought through my opening, which I did not wish to enlarge. This reduction was effected by plunging the scalpel into several of the cysts and evacuating their contents. A gallon or more of fluid was in this way discharged. The tumor was now squeezed through the opening, and but two adhesions were found—*one*, a band of false membrane extended from the side of the fold peritoneum that holds

* Repeated tappings tend greatly to embarras the operation of extirpation, by causing adhesion of the tumors to the walls and organs. This is my experieuce in the former cases. My successful case was tapped seventeen times.

the sigmoid flexure in its place to the side of the tumor near its top, adhering only by a surface as large as the end of the finger. The band was about five inches long, one and a half wide at base, a half inch at tip. The tumor had evidently contracted an adhesion to this reflexion of peritoneum in its ascent from the pelvis, and stretched the false membrane in its further ascent into the abdomen. This false membrane was colorless, (devoid of red vessels,) analogous to cellular tissue, as is always the case in those of ancient formation. I have thus minutely described this structure, because it is one of only *three* that were cut within the cavity, and the only cut surface within it that was not tied. It was clipped loose from the tumor, and after examination to see that no blood was escaping from it, it was suffered to pass back into the cavity.

The *other* adhesion to the tumor was the fimbriated extremity of the left fallopian tube, which was spread out on the side of the tumor, much after its fashion when embracing a graffian vesicle. The whole extremity of the tube was quite red, and when dissected from the tumor, exhibited an extensive irregular surface from which the blood *oozed* freely. I put a strong ligature around the tube itself one and a half inches from the uterus, and cut it off. No farther escape of blood took place. The *pedicle* of the tumor was now its only remaining attachment—this was something over an inch wide—I put a strong silk cord around it, and tied it very firmly. The pedicle was separated and the tumor removed. Not a drop of blood escaped from the pedicle, and I dont think that more than three ounces were lost during the operation. After I had satisfied myself that there were no *bleeding vessels* any where, I asked Dr. Wood, of Liberty, (formerly of St. Louis,) and another old physician, to examine if there was any? They did so, and reported none. (I was thus particular, because the case looked so favorably, that I wanted to make it absolutely certain of a successful result.) The incision was now closed by a number of twisted sutures, adhesive strips compressed and a parturient bandage. The pulse continued good during the operation, which lasted eighteen minutes according to a bystander; and the effects of the chloroform being allowed to subside, the patient waked up and expressed herself as feeling comfortable. She had suffered no pain and would not believe that the operation had been performed. In half an hour she was removed to bed, and said she felt no inconvenience except soreness. The operation was completed half past 12, P. M. In an hour and a half after the operation she had *slight* rigors that lasted a few minutes. Re-action came on but it was moderate, pulse about 100, but soft and compressible. There was no pain, no heat of skin, but there was excessive thirst. Cold water and ice were allowed, but as soon as any accumulation took place in the stomach it was thrown up. The *thirst* was the only unpleasant condition of the patient, and towards night this abated somewhat. I staid with her until bed-time, when the thirst had lessened, and she was otherwise doing well. I ordered morphine, and left her in charge of the family physician and another intelligent young physician ; the latter of whom, a relative of the patient, staid in the house.

At half past two, a messenger brought me word that the patient had not slept; in other respects as I had left her. I directed the morphine repeated, to produce sleep. One portion had the effect—and at half past three, the young doctor (who staid with her) went to bed, leaving her asleep, pulse good, and, as he thought, in better condition than at any time since the operation.

At six o'clock, the family physician (who was up with an obstetrical case) saw her, and found her sleeping so quietly, that he made no examination of the pulse, for fear of disturbing her. At day-light, I went to see her, and found her pulseless or nearly so, and with hurried, difficult respiration, and great distress. She was manifestly sinking very rapidly. What was the cause? This question was necessary to any treatment. It surely was not the shock of the operation, for that was slight, and had long since, passed off. There had been no symptoms of inflammation, and it was much too soon for it to have completed its work. *Hemorrhage* was the only remaining possible cause. It would not do to open the wound to see if my ligatures had slipped, without first bringing the pulse up by stimulants. They were freely used, but produced no effect, and at 9 o'clock, (twenty and a half hours after the operation,) she died.

On examination, I found about two quarts of blood in the abdomen—revealing the cause of her death. *But my*

*ligatures were in place*, and not a drop of blood could h[illegible] escaped by them.

What was the source of this hemorrhage? It cou[illegible] not have taken place within the cavity; for the only stru[illegible]tures cut, were the pedicle, the fallopian tube, and th[illegible] band of false membrane. The ligatures were found firmly applied to the two former, and the latter was a bloodless structure, which could not possibly have furnished it. The only other possible source was the edges of the incision through the abdominal walls. Besides this conclusion, which we arrive at, by the method of exclusion, the conditions of the lips of the wound, at the *post mortem*, *positively* indicated it. *They were very loose and flabby.* If the mouths of the vessels had been plugged up, the edges would have been turgid and firm.

This was surely a most extraordinary result. The abdominal walls were attenuated from long continued distension, and the cut surface was small. I should remark, that my incision deviated about one half an inch to the left of the median line, and took off a narrow strip of the left rectus muscle leaving it attached to the right border of the incision. But neither here nor in the whole length of the incision was there a vessel cut large enough to cause even the smallest arterial jet, none that any surgeon would think of tying in any operation. Well, the only precaution which could have been taken to guard against hemorrhage from this source, was to have left the wound open until the edges were glazed with lymph. But there is always an insuperable objection to this in this operation, viz: it would be leaving the abdominal organs too long exposed to the air. The risk of exciting inflammation would be incomparably greater than that from hemorrhage.—*St. Louis Medical and Surgical Journal.*

